# Hiding in plain sight: vesicle-mediated export and transmission of prion-like proteins

**DOI:** 10.15698/mic2020.07.724

**Published:** 2020-06-02

**Authors:** Mehdi Kabani

**Affiliations:** 1 Institut de Biologie François Jacob, Molecular Imaging Research Center (MIRCen), Commissariat à l'Energie Atomique et aux Energies Alternatives (CEA), Direction de la Recherche Fondamentale (DRF), Laboratoire des Maladies Neurodégénératives, Centre National de la Recherche Scientifique (CNRS), F-92265 Fontenay-aux-Roses.

**Keywords:** yeast prions, prion-like proteins, extracellular vesicles, traffic

## Abstract

Infectious proteins or prions are non-native conformations of proteins that are the causative agents of devastating neurodegenerative diseases in humans and heritable traits in filamentous fungi and yeasts. Prion proteins form highly ordered self-perpetuating fibrillar aggregates that traffic vertically and horizontally from cell to cell. The spreading of these infectious entities relies on different mechanisms, among which the extracellular vesicles (EV)-mediated traffic. The prion form of the yeast *Saccharomyces cerevisiae* Sup35p translation terminator causes the [*PSI*^+^] nonsense suppression phenotype. This fascinating biological model helped us shape our understanding of the mechanisms of formation, propagation and elimination of infectious protein aggregates. We discovered that Sup35p is exported via EV, both in its soluble and aggregated infectious states. We recently reported that high amounts of Sup35p prion particles are exported to the yeast periplasm via periplasmic vesicles (PV) in glucose-starved cells. EV and PV are different in terms of size and protein content, and their export is inversely regulated by glucose availability in the growth medium. We believe these are important observations that should make us revise our current view on the way yeast prions propagate. Hence, I propose several hypotheses as to the significance of these observations for the transmission of yeast prions. I also discuss how yeast could be used as a powerful tractable biological model to investigate the molecular mechanisms of vesicle-mediated export of pathological protein aggregates implicated in neurodegenerative diseases.

## PRION-LIKE PROTEINS ARE VECTORS OF HERITABLE TRAITS IN YEAST AND NEURODEGENERATIVE DISEASES IN HUMANS

“Proteinaceous infectious particles”, or prions, are transmissible conformational states of proteins described mostly in mammals, filamentous fungi and yeasts. Bacterial and plant prion-like proteins were also reported, suggesting this phenomenon is universal and widely conserved throughout evolution. The prototypic mammalian prion protein PrP is the cause of infectious diseases, such as scrapie in sheep, bovine spongiform encephalopathy (BSE) in cow, chronic wasting disease in deer and elk, as well as kuru and Creutzfeld-Jacob diseases in humans. Additionally, the prion-like behavior of proteins such as α-synuclein, Tau, amyloid-β or huntingtin is at the origin of devastating neurodegenerative diseases such as Alzheimer's, Parkinson's or Huntington's diseases. The yeast *Saccharomyces cerevisiae* hosts many unrelated prion proteins that are at the origin of heritable phenotypic traits. Of particular importance, decades of investigations into the [*PSI*^+^] nonsense suppression phenotype, which originates from the prion form of the translation terminator Sup35p, allowed tremendous insights into the mechanisms of prion genesis, propagation and elimination. A common feature shared by all these prion proteins is their ability to assemble into structurally distinct and highly ordered self-replicating fibrils, most often of amyloid nature, that spread vertically or horizontally from cell to cell.

## PERIPLASMIC AND EXTRACELLULAR VESICLES AS VEHICLES FOR YEAST PRION TRAFFICKING

Yeast prions are faithfully transmitted to daughter cells and mating partners via cytosolic diffusible protein entities often referred to as ‘propagons', but these are still not documented at the molecular level. This prompted us to investigate the properties and molecular nature of propagons. We showed that the infectivity of Sup35p prion particles changes dramatically during growth, depending on the metabolic status of the cells. Importantly, we discovered that infectious Sup35p prion particles are packaged within small exosome-like vesicles (~30-100 nm in diameter) and exported in the extracellular space. These extracellular vesicles (EV) are produced by virtually all living cells including cell-walled microorganisms such as yeasts and fungi. EV are thought to play important roles for intercellular communication by acting as vehicles for the transfer of nucleic acids, proteins, signaling molecules, sugars, lipids, and toxins or other pathogenic factors. EV mediate the clearance and contribute to the cell-to-cell transfer of prion-like protein aggregates associated with neurodegenerative diseases. Building upon this initial observation, we found that in glucose limiting conditions, surprisingly high amounts of Sup35p prion particles are exported into the periplasm via periplasmic vesicles (PV) (Kabani *et al.* (2020), Mol Microbiol. doi: 10.1111/mmi.14515). We showed significant differences between PV and EV in terms of size, protein cargo and regulation by glucose availability. However, Sup35p-containing EV and PV probably share a common subcellular origin, and packaging of prion particles within vesicles occurs in actively dividing cells. Using protein transformation assays, we showed that Sup35p prion particles retain their infectivity within EV and PV. Thus, vesicle-embedded prion particles constitute a suitable source of propagons.

## IMPLICATIONS FOR OUR UNDERSTANDING OF YEAST PRION PROPAGATION

**Figure 1:**
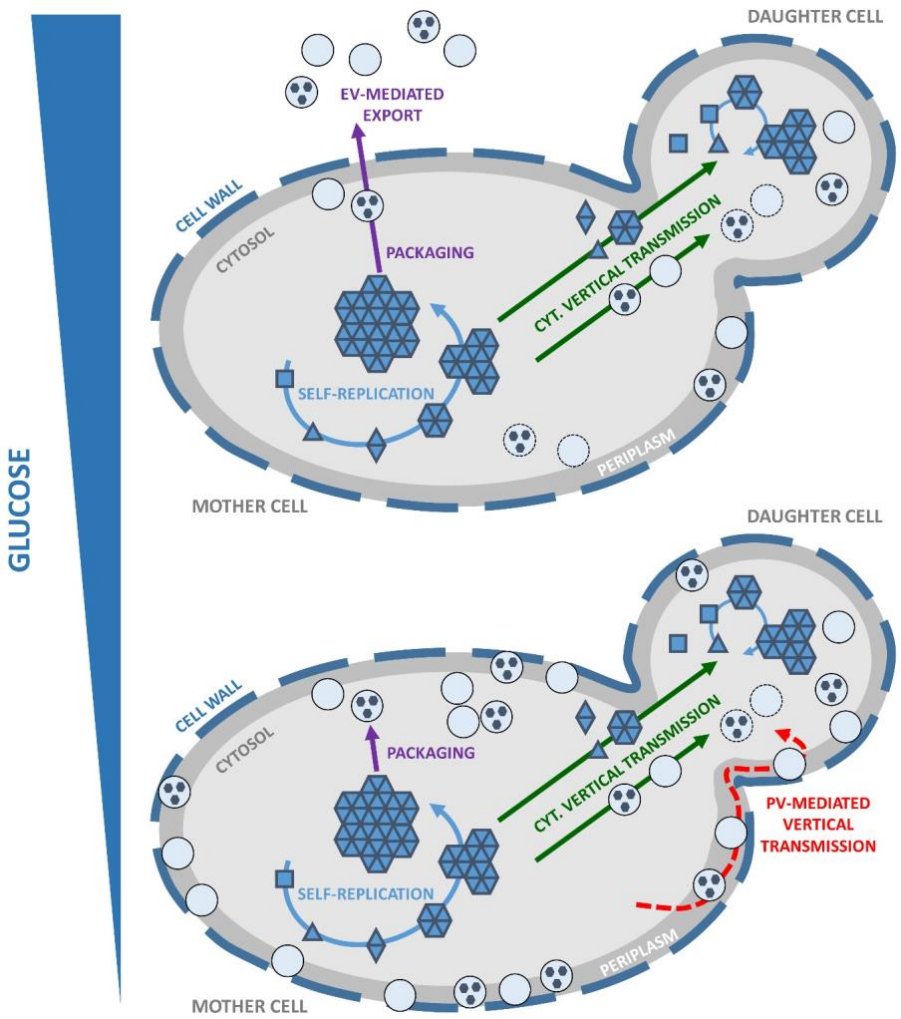
FIGURE 1: Working model for vesicle-mediated yeast prion propagation. Yeast prions self-replicate by the conversion of newly synthesized soluble monomers (squares) to a prion conformation (triangles) and their subsequent incorporation within high-molecular weight prion assemblies (solid blue arrows). Unidentified prion species or ‘propagons' are vertically and cytoplasmically transmitted to the daughter cell (solid green arrows) where they initiate a new round of prion self-replication. Yeast prions are packaged within vesicles in the cytosol (solid purple arrows). Vertical transmission of yeast prions could be mediated by propagons packaged within intracellular vesicles (solid green arrow) transported from mother to daughter cells where their content would be released in the cytosol to initiate a new cycle of prion self-replication. Under high glucose conditions, EV are exported across the cell wall (depicted as a thick dashed line) and to the extracellular medium (*upper panel*; solid purple arrows). Under limiting glucose conditions, high amounts of PV accumulate in the periplasm. Vertical transmission of yeast prions could be mediated by the reinternalization of PV and release of their content in the cytosol of daughter cells (*lower panel*, red dashed arrow).

It appears that a pool of infectious Sup35p prion particles ‘hidden' within vesicles is constituted in actively dividing cells, regardless of whether these vesicles are ultimately exported as EV or PV. We still need to address whether our findings apply to other yeast prions. In the meantime, we propose a working model for the propagation of yeast prions that takes into account their packaging within vesicles, which we believe plays a prominent role (**Figure 1**). The cytoplasmic transmission of yeast prions during cell division is well established yet the precise molecular nature of propagons is unknown. It is also not clear whether the propagons are naked, coated with associated proteins or encapsulated into vesicles. Vesicle-embedded prion particles fulfill the essential features needed to qualify as propagons. First, they are able to faithfully seed homologous monomeric prions and induce the prion state. Secondly, their small size and mobility allow them to traffic efficiently across the bud neck and reach the daughter cell, in line with the high mitotic stability observed for most prion strains. Indeed, secretory vesicles significantly contribute to the growth of the yeast bud where they provide the macromolecules and enzymes required for building the newly forming plasma membrane and cell wall. Finally, propagons are shielded from the action of protein assemblies remodeling activities within the cell, such as molecular chaperones and proteolytic machineries. By hijacking the mother-to-bud vesicular traffic, prion particles could get around spatial quality control, a mechanism of asymmetric retention that protects daughter cells from aging damage accumulated by mother cells. Periplasmic vesicles, which could be reinternalized depending on growth conditions and the metabolic state of the cells, may also efficiently transmit prion particles to daughter cells while avoiding molecular crowding and ‘check-point' controls at the bud neck.

## YEAST AS A MODEL TO INVESTIGATE THE VESICLE-MEDIATED EXPORT OF PATHOLOGICAL PRION-LIKE PROTEIN ASSEMBLIES

Understanding how and when prion-like proteins are selected and packaged inside vesicles is of an interest that surpasses the yeast prion field. Because fundamental cellular functions are conserved from yeast to humans, our work provides an important proof-of-principle for the possible use of yeast as a model to investigate the mechanisms that control the selection, targeting and packaging of prion-like protein aggregates within vesicles. Indeed, misfolded protein assemblies such as α-synuclein or Tau have been found in different types of EV (e.g. exosomes, ectosomes) which have been proposed to act as vehicles for their prion-like spreading pattern in neurodegenerative diseases. When heterologously expressed in yeast, human prion-like proteins and their variants bearing known familial mutations often retained their aggregation and cytotoxic properties. Furthermore, known modifiers of protein aggregation such as molecular chaperones or proteolytic systems are conserved in yeast and are able to act on these prion proteins. Yeast offers a vast array of fast and cost-effective experimental possibilities, including but not limited to the availability of genome-wide deletion and mutant libraries or automated high-throughput drug screening. While the isolation of EV from yeast culture supernatant is a cumbersome process poorly suited for screening purposes, the isolation of PV is on the contrary quick, easy and compatible with high-throughput assays. We predict that developing yeast models of vesicle-mediated prion proteins export will allow us not only to gain fundamental knowledge of the underlying mechanisms but also to design alternative strategies against the propagation of neuropathological protein assemblies.

